# Construction of Novel Bimetallic Oxyphosphide as Advanced Anode for Potassium Ion Hybrid Capacitor

**DOI:** 10.1002/advs.202105193

**Published:** 2022-01-18

**Authors:** Shouzhi Wang, Songyang Lv, Guodong Wang, Kun Feng, Shoutian Xie, Guotao Yuan, Kaiqi Nie, Mo Sha, Xuhui Sun, Lei Zhang

**Affiliations:** ^1^ Institute of Novel Semiconductors State Key Lab of Crystal Materials Shandong University Jinan 250100 P. R. China; ^2^ Suzhou Research Institute Shandong University Suzhou 215123 P. R. China; ^3^ Institute of Functional Nano and Soft Materials (FUNSOM) Jiangsu Key Laboratory for Carbon‐Based Functional Materials and Devices, and Joint International Research Laboratory of Carbon‐Based Functional Materials and Devices Soochow University Suzhou 215123 P. R. China; ^4^ School of Public Administration Shandong Normal University Jinan 250100 P. R. China

**Keywords:** bimetallic oxyphosphide, first principles calculations, in‐situ XRD, integrate anode, potassium ion hybrid capacitor

## Abstract

Potassium ion hybrid capacitors (PIHCs) have attracted considerable interest due to their low cost, competitive power/energy densities, and ultra‐long lifespan. However, the more sluggish insertion kinetics of battery‐type anodes than capacitor‐type cathodes in PIHCs seriously limits their practical application. Therefore, developing advanced anodes with high capacitor and suitable K^+^ intercalation is imperative and significant. A novel core–shell structure of Ni—Co oxide/Ni—Co oxyphosphide (NCOP) nanowires are designed and constructed in this study via efficient and facile strategy. Combining the merits of the core–shell structure and the massive active sites in the oxyphosphide layer, the as‐prepared NCOP composites manifest highly reversible capacitors and outstanding rate capability. Meanwhile, the insertion and conversion potassium storage mechanisms of the NCOP are successfully revealed through in situ X‐ray diffraction and density functional theory calculations, respectively. Furthermore, the PIHC was assembled with NCOP anode and borocarbonitride cathode, which displays a large energy density and high‐power density, along with an exceptional capacity retention of ≈90% over 10 000 cycles at 1.0 A g^−1^. This work provides the anion regulation strategy for modifying the transition metal oxide and constructing the advancing electrode materials for next‐generation energy storage and beyond.

## Graphical abstract

Novel bimetallic oxyphosphide nanowires (NCOP) as advanced anode for potassium ion hybrid capacitor are synthesized via a universal anion‐exchange strategy. Benefiting from the massive defects and multiple cation valence of the oxyphosphide and the stable core‐shell structure of the NCOP, the anode exhibits superior potassium storage capability. DFT calculations and In‐situ XRD reveal the potassium storage mechanism of the NCOP.

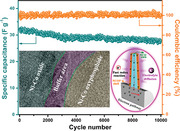

## 1 Introduction

Effective energy storage systems including hybrid/electric vehicles, and an ever‐increasing number of portable electronic gadgets, have a large impact and wide application in the current human life. Among these systems, lithium‐ion batteries (LIBs) with high energy density and supercapacitors (SCs) specializing in power density and ultra‐long cycling performance, have attracted considerable attention. However, with the huge equipment of the device demand for the power and energy density, the LIBs and SCs cannot shoulder this heavy task alone. Alkali‐ion (Li^+^, Na^+^, K^+^) hybrid capacitor, an emerging technologic innovation device, is expected to bridge the secondary battery and SCs to obtain high power and energy densities without sacrificing their performance.^[^ ^1^ ^]^ Potassium ion hybrid capacitors (PIHCs) have attracted research interest due to their natural abundance and lower cost than lithium resources, and lower redox potential of K/K^+^ (−2.94 V versus SHE) than Na/Na^+^ (−2.71 V versus SHE). Furthermore, the stokes radii of the K^+^ ions in PC solution (3.6Å) are smaller than the Li^+^ (4.8 Å) and Na^+^ (4.6 Å), which exhibit the fastest rate of diffusion in the electrolyte solution and the highest molar conductivity.^[^ ^2^ ^]^ Nevertheless, the larger radius of the K^+^ ion (1.38 Å) is prone to cause high diffusion barriers and sluggish ion insertion kinetics in electrodes during the energy storage process.^[^ ^3^ ^]^ Until now, anode materials for potassium ion hybrid capacitor (PIHCs) suffer from either poor cycle life or low energy/power densities. Therefore, exploring suitable anode candidates for high‐performance PIHCs is essential.

Transition metal oxide is a family of materials that have been extensively studied in recent years as the potential anode in the field of potassium storage due to their unique electrochemical properties. Among these materials, binary metal oxides, such as NiCo_2_O_4_, are attracting tremendous interest due to the higher electrochemical activity than single metal oxide, high theoretical capacity benefiting from the multivalent nickel and cobalt, and the multielectron transfer process.^[^ ^4^ ^]^ The shortcomings especially the volume expansion, low conductivity, and side reaction during discharge/charge, are also prevalent, resulting in the rapid decay of capacity.^[^ ^5^ ^]^ Many methods have been performed on size control, structural design, and composite preparation, such as composite graphene or carbon nanotube,^[^ ^6^ ^]^ coated carbon shell.^[^ ^7^ ^]^ Although these strategies could improve the electrochemical performance of metal oxides’ materials to a certain extent, a breakthrough can be also achieved by fundamental studies on the crystallography of materials.

Anion doping in metal compounds is an effective strategy for engineering the properties of nanomaterials.^[^ ^8^ ^]^ Specifically, the introduction of non‐metal anions (N, S, and P) into binary metal oxides (oxynitride,^[^ ^9^, ^10^ ^]^ oxysulfide,^[^ ^11^ ^]^ and oxyphosphide^[^ ^12^ ^]^) can tune their near‐surface electronic structures and increase the exposed active sites, which offers improved charge transfer between different ions and modified electronic structures to decrease the kinetic energy barriers of the electrochemical processes,^[^ ^13^ ^]^ and further enhance the active sites, improving their electrochemical performance.^[^ ^11^ ^]^ Among them, binary metal oxyphosphides have been considered to be one of the most increasing categories in potassium ion storage. The anti‐bonding orbital is depleted with the part replacement of oxygen atoms in NiCo_2_O_4_ by phosphorus atoms due to the few valence electrons of phosphorus atoms, thereby enhancing the metal ligand bond and further improving the stability of the structure.^[^ ^12^ ^]^ Similarly, the adsorption free energy of potassium ions can be adjusted to be thermally neutral via phosphorus doping, thereby enhancing electrochemical activities.^[^ ^13^, ^14^ ^]^ Binary metal oxyphosphide usually involves a multi‐step reaction mechanism as electrode for electrochemical energy storage.^[^ ^15^, ^16^ ^]^ However, the physicochemical changes of the binary metal oxyphosphide during the potassium ion storage remain unclear, which hinders the design and development of advanced electrode materials. Therefore, exploring the reaction mechanism of metal oxides not only facilitates understanding of the relationship between binary metal oxyphosphide and K^+^ but also achieves a long lifespan, which would be a breakthrough for large‐scale energy storage devices.

A facile strategy for preparing the core–shell structure of Ni—Co oxide/Ni—Co oxyphosphide (NCOP) nanowires as advanced anode in PIHCs is developed in this study. The defect‐modified oxyphosphide layer provides a substantial active site for electrochemical reactions and facilitate excellent ionic/electronic conductivity. The core–shell structure of the NCOP further alleviates the volume expansion during subsequent potassiation/depotassiation. The NCOP anode exhibits superior capacitance and outstanding rate capability due to its unique structure. Moreover, in‐situ XRD and first‐principles’ calculations further demonstrated the reaction mechanisms and the outstanding electrochemical properties of the NCOP electrode. Therefore, the PIHC device combining NCOP anode and borocarbonitride (BCN) cathode delivered a maximum energy density of 166.5 W h kg^−1^ and outstanding capacity retention. This finding suggests that the tuning intramolecular charge transfer between the anion and cation is a novel method to improve electrochemical energy storage.

## 2 Results and Discussions

**Figure 1:**
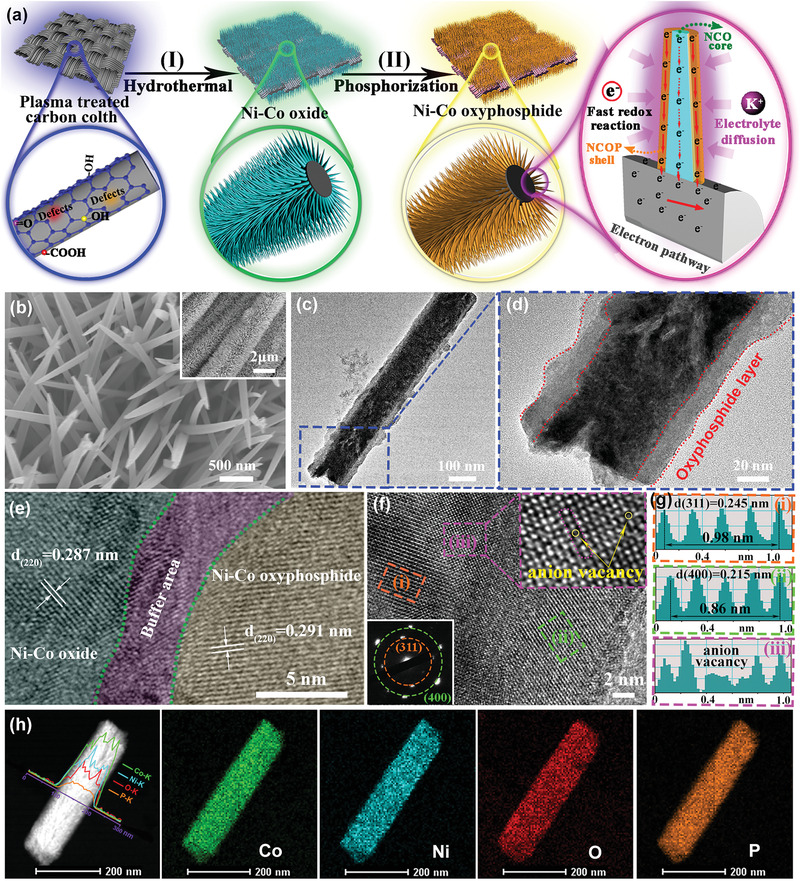
a) Schematic illustration of the synthesis procedure of Ni—Co oxyphosphide samples; b) FESEM image and inset SEM image of the NCOP; TEM image (c) and enlarge image (d); HRTEM image of Ni—Co oxide/Ni—Co oxyphosphide with clear buffer area (e); HRTEM image of NCOP layer and corresponding SAED pattern (f), g) the intensity profiles calculated the interlayer spacing from image (f); HAADF‐STEM image with EDX line‐scan profiles (h) and corresponding element mappings.

The synthetic procedure of the NCOP with core–shell structure is schematically depicted in **Figure** **1**a. Briefly, the Ni—Co oxide precursors were directly grown on the plasma‐treated carbon cloth using the hydrothermal method. Then, the oxyphosphide layer was obtained on the surface of the Ni—Co oxide nanowires and formed the NCOP core–shell structure. In detail, the surface of the air‐plasma‐treated carbon fiber substrate is rougher than the original, as shown in Figures S1a and S1b, Supporting Information. This treatment would cause massive surface defects and oxygen‐containing functional groups on the fiber surface, which can serve as the nucleation site for the precursor crystal seeds during the hydrothermal process.^[^ ^10^ ^]^ The following phosphating process occurs through the anion exchange reaction of the substation of oxygen atoms by phosphorus atoms, leading to the conversion of surface oxides to the oxyphosphide layer. Numerous oxygen defects and the formation of massive multiple valence states are observed in the oxyphosphide layer during this phosphating process, which can be favorable to fast‐redox reactions, further enhancing the electrochemical capacitance. Additionally, the growth of the core–shell NCOP nanowires on the conductive carbon cloth, which can provide an electron highway and shorten the ion diffusion, improves the rate capability and cycling performance.

The morphologies of the obtained Ni—Co based samples were investigated by field‐emission scanning electron microscopy and transmission electron microscopy (TEM). Figure S1c and S1d, Supporting Information, shows the highly uniform growth of as‐synthesized Ni—Co precursor nanowires on the carbon fiber surface. No remarkable changes were found in the morphological characteristics of NCOP after the phosphating treatment (Figure 1b). The TEM image in Figure 1c shows a core–shell structure morphology with a diameter of ≈100 nm. The enlarged TEM image (Figure 1d) shows that the thickness of the shell structure is ≈15 nm, along with some small voids in the core structure. High‐resolution TEM (HRTEM) was investigated for the interface of the core–shell structure (Figure 1e), the inner and outer parts have different lattice orientations, so it could the buffer area in the NCOP. The inner part has lattice fringes with an interplanar spacing of 0.287 nm, which is perfectly assigned to the (220) plane of spinel NiCo_2_O_4_.^[^ ^10^, ^11^ ^]^ Interestingly, the lattice fringes in the outer layer (0.291 nm) are larger than those in theoretical interplanar spacing (JCPPDS#20‐0781) after the replacement of oxygen atoms by phosphorus atoms, thus revealing that the oxyphosphide layer with wide spacing is beneficial for the potassium ion storage. This result further indicates the formation of the NCOP core–shell structure, and the significant increase in the interlayer spacing in the oxyphosphide layer, which could effectively reduce the migration energy barrier of K^+^ intercalation and diffusion, thus further benefiting for potassium ion storage.^[^ ^17^ ^]^

Similarly, the HRTEM image of the oxyphosphide layer also proves this phenomenon (Figure 1f). Figure 1g shows the original intensity profiles from Figure 1f, the well‐defined lattice fringes with a lattice spacing of 0.245 nm in the region (i), which is higher than the standard (311) plane of the NiCo_2_O_4_ (0.244 nm). Furthermore, the significantly expanded interlayer spacing (0.215 nm) of (400) plane is originally observed from the region (ii), which is also larger than the standard value (0.203 nm). This nanostructure in the oxyphosphide layer was effectively supported by the sharp selected‐area electron diffraction spots (Figure 1f inset). More importantly, the region (iii) of Figures 1f inset and 1g reveal the disappearance of several anions from the lattice structure, which directly proves the successful in‐situ introduction of anion vacancies during the phosphating process. The existence of anion vacancies in the oxyphosphide layer could produce additional active sites for K^+^ adsorption and regulate the electronic structure and interface properties of the NCOP.^[^ ^18^ ^]^ Additionally, the high‐angle annular dark‐field scanning TEM and the corresponding elemental energy‐dispersive X‐ray (EDX) line‐scan profiles (Figure 1h), along with the mapping signals, also reveal the uniform distribution of nickel, cobalt, oxygen, and phosphorus elements throughout the entire architecture. This finding is consistent with the SEM mapping result of the NCOP (Figure S2, Supporting Information), which further indicates the formation of NCOP nanowires. The atom ratio of oxygen and phosphorus in NCOP is ≈3:1, which is consistent with the inductively coupled plasma optical emission spectroscopy (ICP‐OES) test result (Table S1, Supporting Information), suggesting the composition of NCOP to be NiCo_2_O_3_P. For comparison, Ni—Co oxide (NCO) and Ni—Co phosphide (NCP) have also been obtained from the Ni—Co precursors, except for modified the annealing conditions, and the relevant SEM images are shown in Figure S3, Supporting Information.

**Figure 2:**
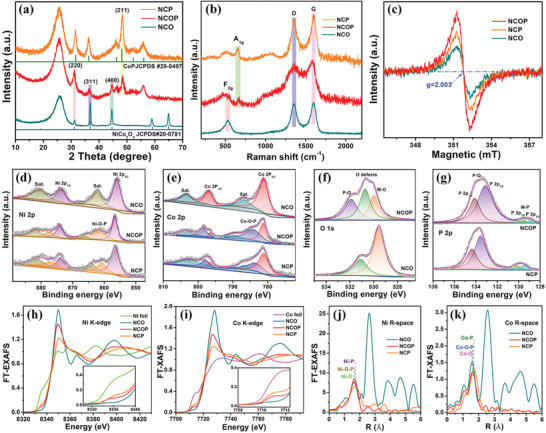
Chemical composition characterization of the Ni—Co oxyphosphide based materials; a) XRD patterns; b) Raman spectra, EPR spectra (c); high‐resolution XPS spectra of Ni 2*p* (d), Co 2*p* (e), O 1*s* (f), and P 2*p* (g); the Ni (h) and Co (i) K‐edge extended XANES spectra and the Fourier transforms at the R space of Ni (j) and Co (k) spectra.

X‐ray diffraction (XRD) patterns were investigated to obtain further insight into the crystallographic structures of the as‐prepared Ni—Co based samples. **Figure** **2**a shows that the diffraction peaks at 31.15° and 44.62° respectively correspond to the (220) and (400) crystal planes of the NCOP sample, which are indexed to the standard structure of NiCo_2_O_4_ (JCPDS#20‐0781) with space group F3dm. Interestingly, the diffraction peaks of NCOP became slightly broader compared with that of the NiCo_2_O_4_, indicating the crystallinity degradation and defect formation after the thermal and phosphating treatment process.^[^ ^19^ ^]^ Particularly, the (311) plane of the NCOP slightly shifts toward the low value of the theta, that is, the wide interplanar spacing of the oxyphosphide layer, which is consistent with the results of HRTEM (Figure 1f). These characteristics could provide a buffer volume fluctuation during the fast potassiation and depotassiation processes of potassium ions.^[^ ^20^ ^]^ The prominent diffraction peak of NCP and NCOP at 48.24° could also be indexed to (211) planes of the standard cubic CoP phase (JCPDS # 29–0497), which indicated that the crystal structure of NCP is similar to that of the cubic CoP phase.^[^ ^21^ ^]^

The Raman spectrum of the NCOP (Figure 2b) showed the presence of distinct peaks at 531 and 666 cm^−1^, which could be respectively ascribed to the F_2g_ stretching modes of the NCO, and A_1g_ vibrational modes of the NCP.^[^ ^22^ ^]^ The bulge peaks at 531 cm^–1^ also appears in the spectrum NCP spectrum, which indicated that seldom NCO contain in the NCP sample. Moreover, a pair of peaks at ≈1350 and ≈1600 cm^−1^ appeared in three Ni‐Co‐based samples, which respectively correspond to the disorder‐induced D band and sp2‐graphitic band of graphene.^[^ ^23^ ^]^ Figure 2c shows that electron paramagnetic resonance (EPR) analysis was conducted to verify the surface defect and vacancy features in the as‐prepared samples. The NCOP displayed a stronger signal than that of the NCO and NCP samples, confirming the presence of additional oxygen vacancies in the NCOP.^[^ ^24^ ^]^ Furthermore, the high oxygen vacancy content in NCOP nanowires will endow the electrodes with excellent electrochemical performance.^[^ ^10^ ^]^

The elemental composition and surface oxidation states of the as‐prepared sample were investigated by X‐ray photoemission spectroscopic (XPS) analysis. The nickel, cobalt, oxygen, phosphorus, and carbon elements were detected in the NCOP products by the XPS survey spectra (Figure S4a, Supporting Information), which is consistent with the EDX results (Figure 1h). The existing C═O bond in the C 1s XPS spectrum (Figure S4b, Supporting Information) ascribes the air‐plasma treated carbon fiber.^[^ ^9^ ^]^ The high‐resolution spectra Co 2*p* (Figure 2d) and Ni 2*p* (Figure 2e) spectra of the NCO, NCOP, and NCP samples were fitted with two pairs of solid state redox couples along with shakeup satellites. In addition, the extra Ni—O—P and Co—O—P characteristic peaks in the intermediate position of the Co^2+^/Co^3+^ and Ni^2+^/Ni^3+^ redox couples in the NCOP could be observed in the Ni 2*p* and Co 2*p* spectra, respectively.^[^ ^25^ ^]^ The Co^2+^/Co^3+^ and Ni^2+^/Ni^3+^ characteristic peaks of the NCOP and NCP have shifted positive compared to that of the NCO, which may be due to the phosphating reaction causes the bond energy change. These results suggest the formation of NCOP during the phosphidation process.^[^ ^9^ ^]^ The three oxygen peaks of the NCOP with binding energies of 531.8, 530.7, and 529.9 eV (Figure 2f) are correspondingly assigned to O—P bonding, oxygen defects, and M—O bonding, and the defects are also consistent with the HRTEM (Figure 1f) and electron paramagnetic resonance (EPR) results (Figure 2c). Furthermore, the Fourier transform infrared spectra (Figure S5, Supporting Information) further confirmed the presence of the Ni/Co—O—P (955.8 cm^−1^) in the NCOP.^[^ ^12^ ^]^ In the P 2*p* spectrum (Figure 2g) of the NCOP and the NCP, the doublet peaks represent P 2*p*_3/2_ (133.1 eV) and P 2*p*_1/2_ (134.1 eV) belonging to P—O bonds, while the peak at 128.9 and 129.6 eV is due to M—P bonds.^[^ ^26^ ^]^ These results indicate the formation of the Ni—Co oxyphosphide and the successful realization of the anion regulation of oxygen and phosphorus at the atomic level.^[^ ^27^ ^]^

The local chemical environment and electronic structure of the Ni—Co based samples are performed from X‐ray absorption near‐edge structure (XANES) spectroscopy analyses. Ni K‐edge spectra at the peak of 8340 eV (Figure 2h) suggest that the valence of nickel in NCOP is higher than that of nickel foil and NCP (Ni^2+^), but lower than that of NCO (Ni^3+^).^[^ ^21^ ^]^ Similarly, the valence of the cobalt in NCOP is between NCP (Co^2+^) and NCOP (Co^3+^) in the Co K‐edge spectra (Figure 2i), which indicated the multiple valence states of the nickel and cobalt in the NCOP samples.^[^ ^4^ ^]^ The distinct change can be further evidenced by the corresponding Fourier transform profiles examined by EXAFS spectra, and the Ni R‐space of the NCO, NCOP, and NCP is shown in Figure 2j. The dominant peak center of the NCOP at the middle of the NCO and NCP samples corresponding the bond lengths of the Ni—O—P bonds (1.64 Å) in the NCOP is large than that of the Ni—O bonds (1.62 Å) in the NCO and shorter than that of the Ni—P (1.70 Å) bonds in the NCP. Similarly, the Co R‐space in Figure 2k shows that the bonding length of the Co—O—P (1.61 Å) in the NCOP is between the Co—P (1.69 Å) and Co—O (1.56 Å). These results demonstrate the mutual interaction between the oxygen and phosphorus centers in the NCOP hybrid, and further indicate the substitution of oxygen for phosphorus at the atomic level. These results agree well with HRTEM and XPS results, indicating the formation of the oxyphosphide layer, regulating the electronic structures, which is critical to the electrochemical redox reaction, and further benefits the electrochemical energy storage.^[^ ^21^ ^]^

**Figure 3:**
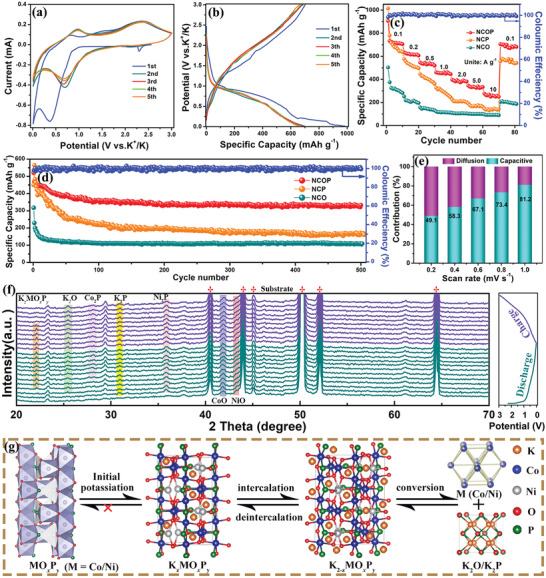
Electrochemical properties of NCOP, a) CV curves of the NCOP electrode at a scan rate of 0.1 mV s^−1^; b) charge–discharge profiles of the NCOS at 0.1 A g^−1^; c) rate performance at various current densities; d) cycling performance at 1 A g^−1^; e) contribution ratios of the capacitive process at different scan rates; f) in‐situ XRD patterns of NCOP anode during potassiation/depotassiation process; g) schematic illustration of the potassium storage mechanism of the NCOP during the energy storage process.

The potassium storage performance of the Ni‐Co‐based electrodes was investigated as self‐supporting anode materials in a potassium half‐cell with a voltage range of 0.01–3.0 V. **Figure** **3**a shows the cyclic voltammetry (CV) profiles of the NCOP electrode at 0.1 mV s^−1^. The pronounced peaks at 2.35, 1.35, and 0.48 V were observed in the first cycles, and then disappeared in subsequent cycles due to K^+^ insertion into NCOP lattice to form different K^+^ contain phases, the formation of a solid electrolyte interphase (SEI) on the electrode surface, and the decomposition of electrolytes. CV profiles remarkably displayed near superposition with each other in the following cycles, implying good reversibility and stability of the NCOP electrode during repeatable potassiation/depotassiation. Figure 3b shows the charge–discharge profiles of NCOP anode at 100 mA g^−1^. The low initial CE (67.8%) is relative to the irreversible solid electrolyte interphase (SEI) and the reconstruction of the irreversible phase during potassium ion intercalation.^[^ ^28^ ^]^ The platform from the charge–discharge profiles is consistent and matches well with the peaks in the CV profiles. Furthermore, the CV curves and charge–discharge profiles of the NCO and NCP samples were investigated as shown in Figure S6, Supporting Information. Compared with the NCOP electrode, the low specific capacity and poor rate capability of the NCO and NCP electrode are attributed to the fragile structure and poor conductivity of the relevant Ni—Co oxides and phosphate.

The rate performances of all the Ni‐Co‐based samples were assessed under various current densities ranging from 0.1 to 10.0 A g^−1^ (Figure 3c). The Figure 3c shows that the specific capacities of the as‐prepared NCOP are 721.5, 629.8, 541.5, 456.4, 395.2, 339.5, and 265.7 mA h g^−1^ at current densities of 0.1, 0.2, 0.5, 1.0, 2.0, 5.0, and 10.0 A g^−1^, respectively. Furthermore, the bare carbon cloth substrate shows a smaller current response than the Ni‐Co‐based material (Figure S7, Supporting Information), which response indicates the negligible contribution of carbon cloth to capacitance.^[^ ^9^ ^]^ All these results are substantially better than those of NCO and NCP, especially under high current densities. Notably, the NCOP electrode could quickly resume a reversible capacity when the current density returns to 0.1 A g^−1^, and the capacity is almost completely recovered (703 mA h g^−1^), suggesting an excellent performance rate of the NCOP. Compared with other anode materials of TMOs for potassium ion batteries, the as‐prepared NCOP displayed a high specific capacity, as shown in Table S1, Supporting Information. The outstanding performance rate of NCOP is mainly ascribed to the oxyphosphide layer and the chemical interfacial interactions toward robust structure and fast electron and ion transport.^[^ ^29^ ^]^

Figure 3d shows the cycle performances of the potassium ion battery with the NCO, NCOP, and NCP electrodes at a current density of 1.0 A g^−1^. The three cycle curves of the NCOP are remarkably steady with no fluctuation after the second cycle and retain capacities of 332.5 mA h g^−1^ after 500 cycles. In addition, almost no changes were observed in the SEM images of the 500 cycles and the initial electrode (Figure S8, Supporting Information). The specific capacities of the NCO and NCP anode are only maintained at 108.2 and 160.5 mA h g^−1^ after 500 cycles, respectively. The capacity of the NCO and NCP electrode rapidly decays at the beginning and then stabilizes, which may be ascribed to the structural collapse of the electrode material during the charging and discharging processes.^[^ ^10^ ^]^ Moreover, even at low current density (0.1 Ag^−1^), the NCOP also maintains 603 mAh g^−1^ after 100 cycles (Figure S9, Supporting Information). These results indicate that the NCOP anode with the unique core–shell structure possesses excellent electrochemical performance and cycle stability.^[^ ^28^ ^]^

The electrochemical kinetics of the NCOP anode was investigated by CV analysis with the scan rates from 0.1 to 1.0 mV s^−1^. As shown in Figure S10a, Supporting Information, the CV curves in the NCOP anode with similar peak positions gradually broadened with the increasing scan rate, indicating the existence of pseudocapacitive behavior during the potassium storage occurrence. The general method was employed on the basis of the following equation to further analyze the degree of pseudocapacitive effect: *i* = a*v* ^b^, where a and b are constants. The b‐value of 1.0 generally indicates a surface‐dominated charge‐storage process, while that of 0.5 represents the K^+^ diffusion‐controlled intercalation process. As expected, the b‐value of the NCOP was ≈0.8 (Figure S10b inset, Supporting Information), suggesting the excellent surface‐dominated characteristics and allowing for fast potassium ion insertion and extraction. Moreover, the detailed contributions of the surface capacitive could be separated in accordance with the following equation: *i* = k_1_ *^*ν*^* + k_2_ *ν* ^1/2^, where k_1_ *ν* represents the surface process contribution, and k_2_ *ν* ^1/2^ indicates the diffusion‐dependent intercalation process. The capacitance contribution of the NCOP is 73.4% at a scan rate of 0.8 mV s^−1^ (Figure S10b, Supporting Information). The capacitive contribution gradually increased from 49.1% to 81.2% with the rising scan rates (Figure 3e), demonstrating that the massive defect sites in the surface of the NCOP materials are beneficial to the fast potassium ion adsorption and further improve the rate capability performance.

Operando XRD was further applied to provide insights into the NCOP electrochemical reaction mechanism during the potassiation/depotassiation process. Figure 3f presents the in‐situ XRD patterns of the NCOP anode, which examined the first cycle accompanied by the initial galvanostatic charge/discharge profiles. The new peaks of K*_z_M*O*_x_* P*_y_* (*M* = Ni/Co) were detected at 22.3° during the discharge process, and the peak gradually weakened with the K^+^ deintercalation, indicating the reversibility of such an intercalation mechanism. The relevant reaction mechanism of the intercalation stages of evolution can be expressed as follows:

(1) $$MO_{x}P_{y}+zK^{+}+ze^{-}↔K_{z}MO_{x}P_{y}\left(M=\mathrm{Ni},\;\mathrm{Co}\right)$$

Two peaks can be observed at 41.8° and 43.2° corresponding to CoO and NiO phase during the first discharge. Simultaneously, four additional peaks were also detected at 25.5°, 28.1°, 30.8°, and 35.8°, respectively corresponding to K_2_O, Co_2_P, K_3_P, and Ni_2_P phases. This finding indicates the exit of K ions from the Ni—Co oxyphosphorus and the occurrence of conversion reaction at this stage. Meanwhile, nanocrystalline nickel and cobalt remained undetected due to their substantially small crystallite size.^[^ ^30^ ^]^ In addition, the intensity of the metal oxide characteristic peaks was stronger than that of the metal phosphate products, thus confirming the dominance of the metal oxide during the potassiation/depotassiation process. The K_3_P and K_2_O peaks were respectively observed in Figure 3f and Figure S11, Supporting Information, indicating the formation of a conversion reaction during potassiation.^[^ ^31^ ^]^ Accordingly, the relevant reversible conversion reaction of NCOP anode may be described by the following equations: first cycle:

(2) $$\begin{array}{ccc} 2MO_{x}P_{y} & + & \left(2x+3y\right)K^{+}+\left(2x+3y\right)e^{-}\to MO_{x}+MP_{y}\\ & + & xK_{2}O+yK_{3}P \end{array}$$

Second cycle and beyond:

(3) $$\begin{array}{ccc} MO_{x} & + & MP_{y}+\left(2x+3y\right)K^{+}+\left(2x+3y\right)e^{-}↔2M\\ & + & xK_{2}O+yK_{3}P \end{array}$$

Figure 3g expresses the intercalation and conversion reactions at molecular‐level structural variation to indicate the reaction mechanism. In initial potassiation, NCOP (*M*O*_x_* P*_y_*) intercalation with K^+^ occurs to form K*_z_M*O*_x_* P*_y_*. In the following conversion reaction, K*_z_M*O*_x_* P*_y_* reacts with K^+^ to generate K_2_O, K_3_P, and *M* (Ni/Co). The metals are then converted to the product of *M*O*_x_* and *M*P*_y_* during charging. The following cycle demonstrates reversible intercalation/deintercalation and conversion reaction respectively between metal oxide/phosphide and K_2_O/K_3_P, which also resulted in the previous report.^[^ ^28^, ^32^ ^]^

**Figure 4:**
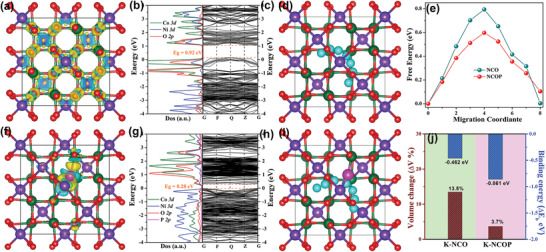
The charge‐density differences of the NCO (a), and NCOP (f), the calculated bandgap structures (b,g) and partial density of states (c,h) for NCO, and NCOP, potassium migration pathway of NCO (d) and NCOP (i), corresponding calculated potassium diffusion barrier in NCO and NCOP (e), color codes: Ni, purple; Co, green; O, red; P, pink; K, cyan; binding energies (ΔEb) for potassium storage in calculated modes, and the volume changes from before to after potassium storage for NCO and NCOP (j).

The first‐principles calculation based on density functional theory (DFT) was performed in **Figure** **4** to clarify the mechanism for the mix‐anions on the electron properties and K^+^ storage performance enhancement of the NCOP. Notably, one oxygen atom was replaced with a phosphorus atom in the NCO unit cell to understand the structure change trend. The crystal structure of the NCOP was almost maintained compared with the NCO, as shown in Figure S12, Supporting Information. However, the charge densities of the NCO (Figure 4a) and NCOP (Figure 4f) have significant differences: the charge around the phosphorus atom increases compared with that of NCO, indicating the electronegativity enhancement of the NCOP and the further improvement of conductivity.^[^ ^11^, ^33^ ^]^ The effect of phosphorus‐doping on the electronic structures was also investigated by the calculated partial density of states (PDOS). Figures 4b,g of the NCO and NCOP reveal that the state density of NCOP displays a slight delocalization after the partial substitution of oxygen atoms by phosphorus atoms. In addition, the partial density of states (PDOS) results show a considerably wide P 2*p* impurity state near the Fermi level, resulting in the lower band gap of NCOP (0.28 eV, Figure 4h) than that of NCO (0.92 eV, Figure 4c). The attraction for 3d electrons of Figure 4g, which mainly comprise Ni 3d, Co 3p, and O 2p orbitals, reveals their intrinsically metallic features and fast rate of charge transfer due to the higher electronegativity and longer bond length of Ni/Co—O—P bonds than those of Ni/Co—O bonds.^[^ ^34^ ^]^

The rate capability is a key parameter that affects the application prospects of battery materials. This parameter is largely affected by the degree of ion migration; that is, ions rapidly migrate when the barrier to overcome is low. The nudge elastic band method of the DFT was engaged to calculate the energy barrier of the K^+^, which can be determined to predict the energy barrier level that must be overcome in each structure. Potassium ions generally migrate between the nearest neighboring sites with the lowest adsorption energy (Eb). Two possible intercalation sites of potassium ions are observed in the NCO crystal lattice based on the calculated adsorption energy (Figure S13, Supporting Information), the adsorption energy in the Ni—Co—O interstitial sites of site 1 (−0.462 eV) is lower than that of Co—O interstitial sites of site 2 (−0.103 eV), as respectively shown in Figure 4j (blue bar) and Table S3, Supporting Information. This finding indicates the preferential insertion of K ions into S1 sites, which are interstitial sites comprising Ni—Co—O.

The possible migration paths of the bulk NCO (Figure 4d) and bulk NCOP (Figure 4i) reveal that the calculated diffusion barrier for the NCOP (0.6 eV) was lower than that of NCO (0.8 eV, Figure 4e), indicating the effective adsorption of K^+^ into the NCOP material and rapid migration through structural experience.^[^ ^35^ ^]^ The volume expansion during the energy storage processes is the main limitation of metal oxides for energy storage applications. DFT calculation was conducted to reveal the aforementioned drawback for metal oxides and oxyphosphorus to determine the volume change before and after K^+^ insertion into the outer surface of the NCO and NCOP, as respectively shown in Figure 4j and Table S3, Supporting Information. The calculation results show that the volume expansions of NCO and NCOP electrode after K^+^ insertion were 13.5% and 3.7%, respectively. This result indicates the stable structure of the NCOP during energy storage,^[^ ^36^ ^]^ which further improved the cycle stability and rate performance of electrode materials.^[^ ^10^ ^]^ Therefore, these results demonstrate the further improvement of K‐ion storage capabilities of the NCOP after the introduction of phosphorus atoms based on the theoretical analysis according to the DFT calculations.^[^ ^37^ ^]^

**Figure 5:**
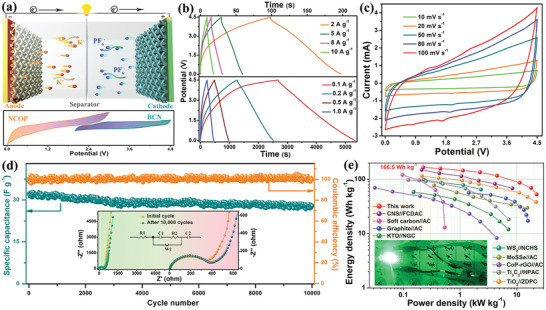
The electrochemical properties of NCOP//BCN PIHC. a) Schematic of the assembled PIHC device (inset shows diagram of the operation potential range for configuration); b) the GCD curves at different current density; the CV curves at increasing scan rate (c); d) The cycling performances at 1 A g^−1^, inset shown the EIS plots of the initial cycle and after 10 000 cycles, and the relevant equivalent circuit diagram; e) Regine plots in comparison with other PIHCs reported in literature; inset shows the image of commercial LED powered by the fabricated PIHC device.

A PIHC device was assembled with NCOP as anode and BCN as cathode to evaluate the potential applications of the NCOP in potassium ion storage. The relevant schematic is illustrated in **Figure** **5**a, for the charging process, the K^+^ cations are intercalated/absorbed into/on the NCOP anode for the charging process, and the PF6^−^ anions are simultaneously absorbed on the surface of the BCN cathode; the discharge process is vice versa. The nanonet morphology, structure, and specific surface area of the BCN for the cathode were respectively investigated in Figures S14–S16, Supporting Information. To characterize the voltage window of the BCN material in the potassium‐ion half‐cell, the linear sweep voltammetry (LSV) curve was engaged in the Figure S17, Supporting Information, from which it can be seen that no polarization phenomenon occurs at 4.8 V and below. Furthermore, the outstanding capacity and the excellent cycling performance of the BCN were displayed from the half‐cell electrochemical performance in Figure S18, Supporting Information. The mass ratio of cathode and anode materials was optimized to 3:1 owing to the electrochemical performance of the as‐prepared PIHCs with the different anode/cathode mass ratios shown in Figure S19, Supporting Information.

As revealed in Figure 5b, the typical GCD curves of the NCOP//BCN device at increasing current densities demonstrated an ultra‐high reversible specific capacity of 59.3 F g^−1^ with a quasi‐linear GCD curve at 0.1 A g^−1^, which can still contribute 21.5 F g^−1^ even at 10 A g^−1^. Notably, the low IR drop of the GCD curves even at high current density reveals the excellent rate capability of the NCOP//BCN system. The superior capacitance can be ascribed to the abundant heteroatom functionalities and surface defects on the NCOP and BCN electrode.^[^ ^38^ ^]^ The CV curve of the PIHCs (Figure 5c) exhibits a quasi‐rectangular shape without polarization and can be operated steadily at an operating voltage of 0.01–4.5 V. This curve further indicates that the storage mechanism is primarily governed by capacitive‐controlled behavior with a small degree of adsorption/desorption.^[^ ^39^ ^]^ Considering cycling stability, the NCOP//BCN device delivers superior cycling stability with a capacity retention of 86.5% after 10 000 cycles at 5.0 A g^−1^ (Figure 5d). The superior rate performance of PIHCs is due to the fast kinetics of the electrode materials. The electrochemical impedance spectroscopy of the devices after 10 000 cycles is compared with the initial cycles, as shown in the Figure 5d inset. In addition, the equivalent circuit is employed to investigate the charge transport kinetics of the devices (Figure 5d inset). The small diameter of the semicircle is related to the synergistic effect of contact resistance (Rs) and charge transfer impedance (Rct) at the interface between electrode and electrolyte. The sloping line is attributed to Warburg impedance (W1), which is connected with the K^+^ diffusion coefficient at the interface of the electrolyte/electrode.^[^ ^40^ ^]^

The energy and power densities of the as‐fabricated NCOP//BCN PIHCs are illustrated in Figure 5e. As expected, the devices deliver an ultrahigh energy density of 166.5 Wh kg^−1^ at 225 W kg^−1^ based on the total mass of active materials in both electrodes. The energy density also remains as high as 53.1 Wh kg^−1^ even at power outputs as high as 22.5 kW kg^−1^. The energy and power densities of these PIHCs are superior to most of the reported state‐of‐the‐art PIHCs in Table S4, Supporting Information, such as CNS//FCDAC,^[^ ^41^ ^]^ WS_2_//NCHS,^[^ ^42^ ^]^ MoSSe//AC,^[^ ^43^ ^]^ KTO//NGC,^[^ ^44^ ^]^ Graphite//AC,^[^ ^45^ ^]^ Soft carbon//AC,^[^ ^46^ ^]^ CoP@rGO//AC,^[^ ^47^ ^]^ Ti_3_C_2_//HPAC,^[^ ^48^ ^]^ TiO_2_//ZDPC,^[^ ^49^ ^]^ MoP@NC‐1//AC,^[^ ^50^ ^]^ and CoV_2_O_6_@GO//AC.^[^ ^51^ ^]^ In addition, a fully charged NCOP//BCN device can easily light up a blue light‐emitting diode (inset of Figure 5e), thus demonstrating its application potential. The current study may introduce a new application field for transition metal oxides and provide an alternative way for the further development of high‐performance PIHCs.

## 3 Conclusion

Overall, a unique core–shell structure of NCOP composite is designed and fabricated through a facile strategy. The NCOP anode delivers outstanding potassium ion storage properties (685.5 mAh g^−1^ at 100 mA g^−1^) due to the unique structural features of the NCOP layer, expanded interlayer spacing, numerous defects, and high‐conductivity carbon fiber networks. Moreover, the in‐situ XRD and DFT calculations are conducted to reveal the relevant electrochemical storage mechanism of the NCOP anode. Taking advantage of the unique structure, high‐performance PIHCs are assembled with the NCOP anode and BCN cathode, thus demonstrating extraordinarily high energy/power densities (166.5 Wh kg^−1^ and 22.5 kW kg^−1^, respectively) and excellent cyclic lifespan. This conception and design of an anion regulation transition metal compound for advanced PIHCs will introduce a novel method for the development of hybrid ion capacitors with high energy/power densities.

## 4 Experimental Section

### Preparation of Ni‐Co‐based composites

cobalt chloride hexahydrate (6 g), nickel chloride hexahydrate (12 g), and urea (2.7 g) were dissolved in 80 mL DI water, then a piece of carbon cloth (2 × 2.5 cm) was put into the above solution. Thereafter, the solution and the treated‐carbon cloth were transferred to a 100 mL autoclave, and heated at 130 °C for 6 h. The as‐prepared precursors on the carbon cloth were washed with DI water and ethanol several times, and dried at 80 °C for 20 h. The as‐prepared Ni—Co precursor was positioned on the top of quartz boat, with 0.5 g of sodium hypophosphite powder at the bottom side. Finally, the quartz boat was place in a tubular furnace and annealed at 350 °C for 1.5 h at a heating rate of 10 °C min^−1^ under a N_2_ flow, and NiCo_2_O_3_P composite (NCOSP) was obtained. For comparison, the Ni—Co precursor was annealed at 350 °C for 2 h in under a flow of nitrogen to obtain a NiCo_2_O_4_ sample (NCO), and the Ni—Co was placed in a quartz with sodium hypophosphite (1.0 g), heated at 350 °C for 4 h to obtain a binary transition metal phosphide sample (NCP). And the precursors were under the same experimental conditions. The average loading density of these active materials was ≈1.0 mg cm^–2^.

## Conflict of Interest

The authors declare no conflict of interest.

## Supporting information

Supporting Information

## Data Availability

The data that support the findings of this study are available from the corresponding author upon reasonable request.
