# Supplementary material for: Construction of Novel Bimetallic Oxyphosphide as Advanced Anode for Potassium Ion Hybrid Capacitor
Source: Adv Sci (Weinh). 2022 Jan 18;9(9):2105193. doi: 10.1002/advs.202105193 (PMC8948644; doi:10.1002/advs.202105193)
Supplement: Supplementary file 1 — Supporting Information [file ADVS-9-2105193-s001.pdf]

ADVANCED SCIENCE
Open Access

Supporting Information

for *Adv. Sci.*, DOI: 10.1002/advs.202105193

Construction of Novel Bimetallic Oxyphosphide as Advanced Anode for Potassium Ion Hybrid Capacitor

Shouzhi Wang[*,a,b,†], *Songyang Lv*[a,†], *Guodong Wang*[a], *Kun Feng*[c], *Shoutian Xie*[d], *Guotao Yuan*[c], *Kaiqi Nie*[c], *Mo Sha*[c], *Xuhui Sun*[*,c] *and Lei Zhang*[*,a]

Supporting Information

Construction of Novel Bimetallic Oxyphosphide as Advanced Anode for Potassium Ion Hybrid Capacitor

Shouzhi Wang[*,a,b,†], *Songyang Lv*[a,†], *Guodong Wang*[a], *Kun Feng*[c], *Shoutian Xie*[d], *Guotao Yuan*[c], *Kaiqi Nie*[c], *Mo Sha*[c], *Xuhui Sun*[*,c] *and Lei Zhang*[*,a]

[a]Institute of Novel Semiconductors, State Key Lab of Crystal Materials, Shandong University, Jinan, 250100, P. R. China.

[b]Suzhou Research Institute, Shandong University, Suzhou, 215123, P. R. China.

[c]Institute of Functional Nano and Soft Materials (FUNSOM), Jiangsu Key Laboratory for Carbon-Based Functional Materials and Devices, and Joint International Research Laboratory of Carbon-Based Functional Materials and Devices, Soochow University, Suzhou, 215123 P. R. China.

[d]School of Public Administration, Shandong Normal University, Jinan, 250100, P. R. China.

[*]E-mail: wangsz@sdu.edu.cn; leizhang528@sdu.edu.cn; xhsun@suda.edu.cn

[†]These authors contributed equally to this work.

This file includes:

S1. Materials

S2. Experimental Section

S3. Characterization Methods

S4. Electrochemical Measurements.

S5. Calculation of Capacitance Contribution

S6. Supplementary Figures S1–S15

S7. Table S1–S3

S1. Materials

All the reagents were purchased from Sinopharm Chemical Reagent Co., Ltd. (Shanghai). All the reagents were used as received.

S2. Experimental Section

S2.1 Preparation of BCN: Firstly, boric acid (6 g), urea (3 g), and PEG-2000 (0.3 g) were dissolved in 60 mL deionised water under stirring for 15 minutes. Then, the air plasma treated carbon cloth (1.5 cm ×3 cm) was immersed in the above solution. The solution including the carbon cloth in a beaker were heat at 85 ℃ for 12 h. Finally, the precursor was pyrolysed at 950 ℃ for 5 h under Ar atmosphere and the BCN sample was obtained. To better match the anode material and optimize the performance of the PIHC device, the addictive amount of PEG-2000, boric acid and urea in the precursors was appropriately increased to enhance the mass loading.

S2.2 Theoretical calculation method: We have employed the first-principles to perform all Spin-polarization density functional theory (DFT) calculations within the generalized gradient approximation (GGA) using the Perdew-Burke-Ernzerhof (PBE) formulation. We have chosen the projected augmented wave (PAW) potentials to describe the ionic cores and take valence electrons into account using a plane wave basis set with a kinetic energy cutoff of 450 eV. Partial occupancies of the Kohn−Sham orbitals were allowed using the Gaussian smearing method and a width of 0.03 eV. The electronic energy was considered self-consistent when the energy change was smaller than 10^{-6} eV. A geometry optimization was considered convergent when the energy change was smaller than 0.03 eV $Å^{-1}$. The Brillouin zone integration is performed using

3×3×3 Monkhorst-Pack k-point sampling for a structure. Finally, the adsorption energies (Eb) were calculated as Eb= Ead/sub -Ead -Esub, where Ead/sub, Ead, and Esub are the total energies of the optimized adsorbate/substrate system, the adsorbate in the structure, and the clean substrate, respectively. For Co and Ni atom, the U correction has been employed in our systems. Finally, the K ions migration barrier energies had been evaluated using the climbing nudged elastic band (CI-NEB) methods.

S2.3 Fabrication of the potassium ion half-Cell and Potassium Ion Hybrid Capacitors: The NCOP materials and BCN were directly used as self-supporting electrode. The potassium ion half-cell was assembled into coin-type test cell (CR2032) with potassium metal working as both the counter and reference electrode. The electrolyte was 1 M KPF_6 in in ethyl carbonate)/dimethyl carbonate (DMC) + fluoroethylene carbonate (FEC) (EC:DMC, 1:1 by volume ratio, 5 *wt*% FEC) and glass microfiber filter as the separator. The PIHC device is composed of NCOP anode and BCN cathode in a glove box filled with argon. Note that the electrode was pre-potassiated by directly contacting with potassium metal in the electrolyte before assembling the device.

S3. Characterization Methods

Scanning electron microscopy (SEM) images were obtained using a Hitachi S-4800 field emission microscope. Raman spectra of samples were obtained with a LabRAM HR system of Horiba Jobin Yvon at room temperature and using a 447 nm solid state laser as the excitation source. XRD was collected by a Bruker D8 Advance X-ray diffractometer with Cu Kα radiation (λ = 1.5406 Å). High-resolution

transmission electron microscopy (HRTEM) images were obtained on a Philips Tecnai Twin-20U high-resolution transmission electron microscope that operates at an accelerating voltage of 200 kV. The EPR spectra were obtained using a JEOL (FA200) EPR spectrometer at X-band (≈ 9 GHz) at 123.15 K. The Co K-edge and Ni K-edge X-ray absorption near-edge spectra (XANES) were performed at the BL14W1 beamline of Shanghai Synchrotron Radiation Facility (SSRF). The surface area was determined by Brunauer–Emmett–Teller (BET) measurements with an ASAP 2020 sorptometer. PSD was determined according to Barrett–Joyner–Halenda (BJH) desorption d*V*/dlog(*r*) plot volume for the mesoporous, and the microporous was determined by H-K method.

S4. Electrochemical Measurements.

Electrochemical impedance spectroscopy (EIS) measurements and cyclic voltammogram (CV) measurements were carried out using a CHI 660E electrochemical workstation. Alternating current EIS spectra were collected within a frequency range of 10^{-2} Hz–10^{5} Hz at the open circuit voltage with AC amplitude of 0.005 V. The galvanostatic charge−discharge performance of the half cells was performed on a Neware battery test system, and the galvanostatic charge−discharge performance of the potassium-ion hybrid capacitors (PIHC) was carried out using a CHI 660E electrochemical workstation. All of the electrochemical tests were implemented at room temperature.

Specific capacitance (F g^{-1}) of the PIHC device was calculated through the following equations:

$$C_{cell} = \frac{I\Delta t}{m\Delta V} \quad (1)$$

where I is the constant discharge current (A), Δt is the discharge time (s), ΔV is the voltage window (V), m is the total mass loading of the active material on the two electrodes (mg).

The energy density (E) and power density (P) of the SIC were estimated according to Equation 3-5, respectively, represented as:

$$P = \Delta V \times i/m \quad (2)$$

$$E = P \times t/3600 \quad (3)$$

$$\Delta V = (V_{max} + V_{min})/2 \quad (4)$$

where i (A), m (g), and Δt (s) denote the discharge current, mass of active materials, and discharge time, respectively. V_{min} (V) and V_{max} are the minimum and maximum working voltage during the charge-discharge process, respectively.

S5. Calculation of Capacitance Contribution

The b value from $i = av^{b}$ can be obtained by plotting current and sweep rate in logarithm, namely, the gradient of linear plots.

$$\log i = b \log v + \log a \quad (5)$$

In reality, the current can originate from both aforementioned contributions. Therefore, the b value can vary between 0.5 and 1. Taking that into consideration, we can rewrite Equation (6) so that the current is a sum of two parts: capacitive current (viz., b=1) and ion-diffusion controlled one (viz., b=0.5):

$$i(V) = k_1 v + k_2 v^{1/2} \quad (6)$$

We are interested in the percentage of the capacitive current. So, in order to determine the $k1$ value, equation (7) can be reformulated as:

$i(V)/v^{1/2}=k_1\, v^{1/2} + k_2$ (7)

Obviously, with a series test of CV curves under different scan speed, the $k1$ value can be determined from the linear plots of $i(V)/\, v^{1/2}$ vs $v^{1/2.}$

S6. Supplementary Figures S1–S15

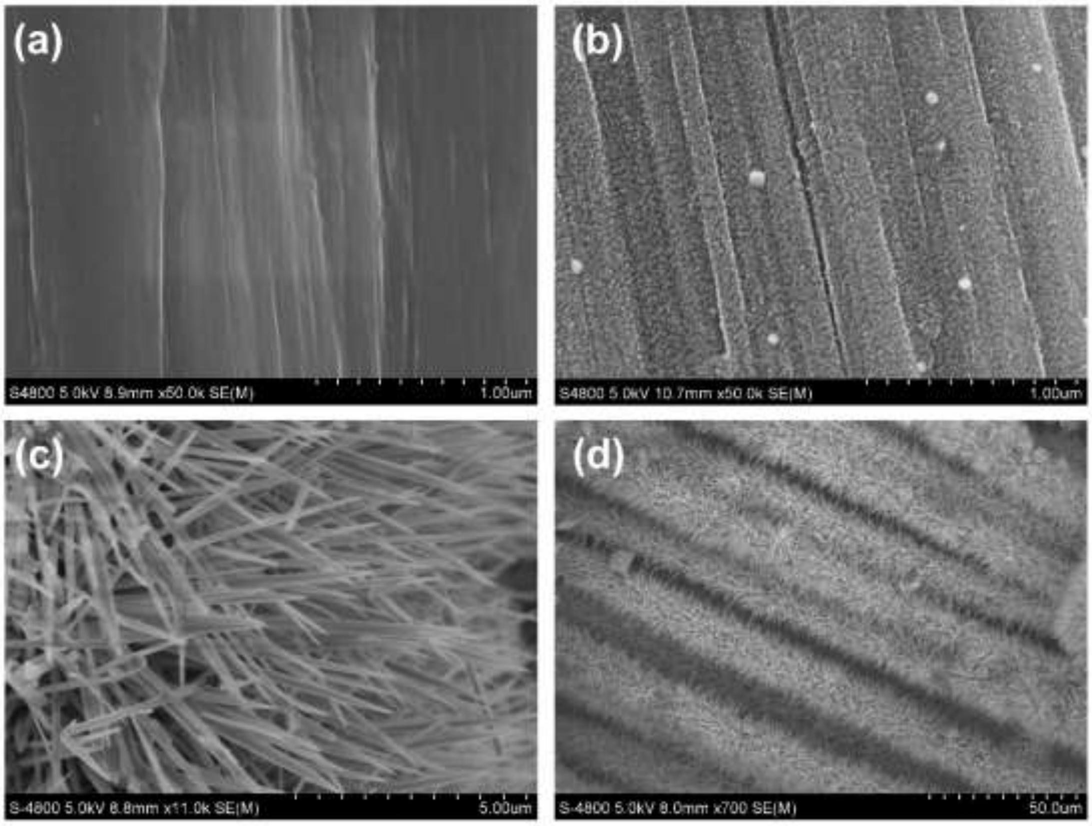

Figure S1. SEM images of the carbon cloth before (a) and after (b) plasma-treatment, and the SEM images of Ni-Co precursors (c-d).

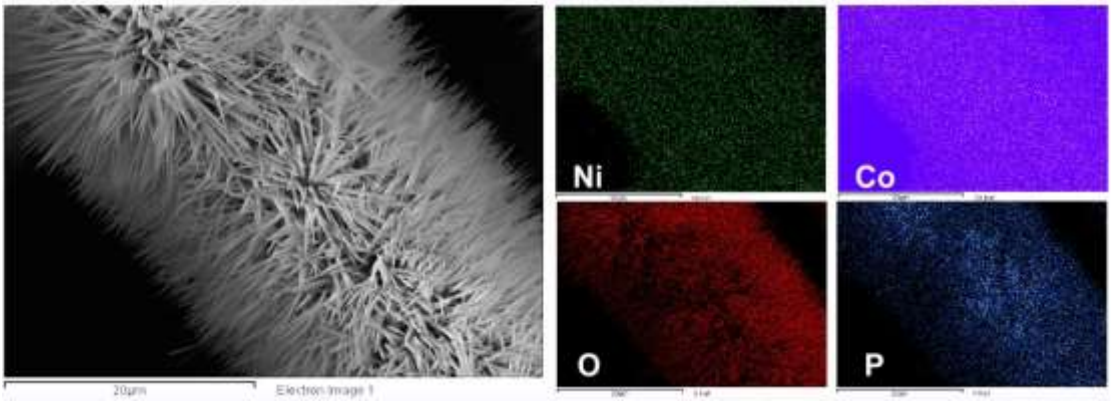

Figure S2. SEM images and the corresponding mapping images of the NCOP samples.

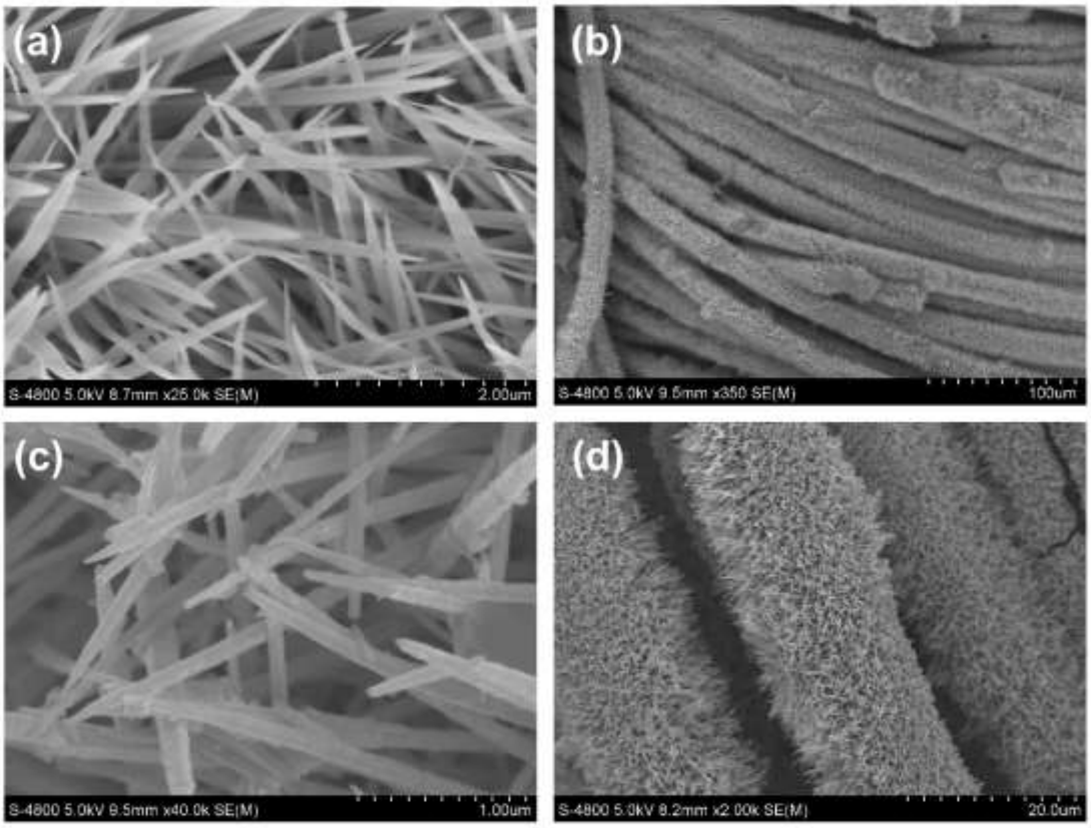

Figure S3. SEM images of the NCO (a, b) and NCP (c, d).

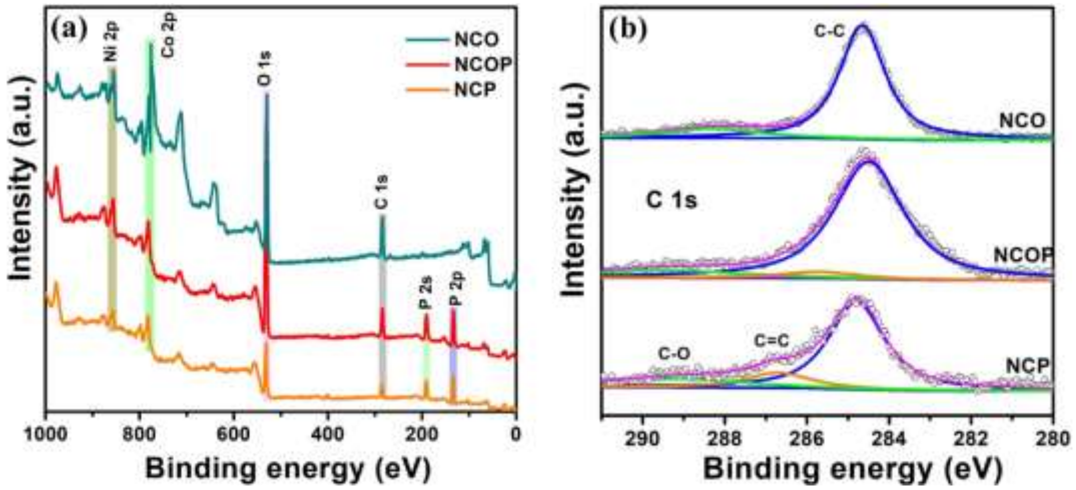

Figure S4. The XPS survey (a) and the C 1*s* spectrum (b) of the Ni-Co based materials.

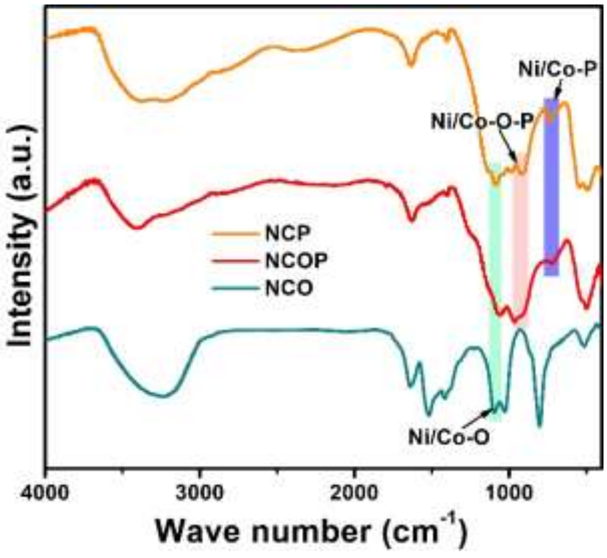

Figure S5. FTIR spectra of the Ni-Co composites

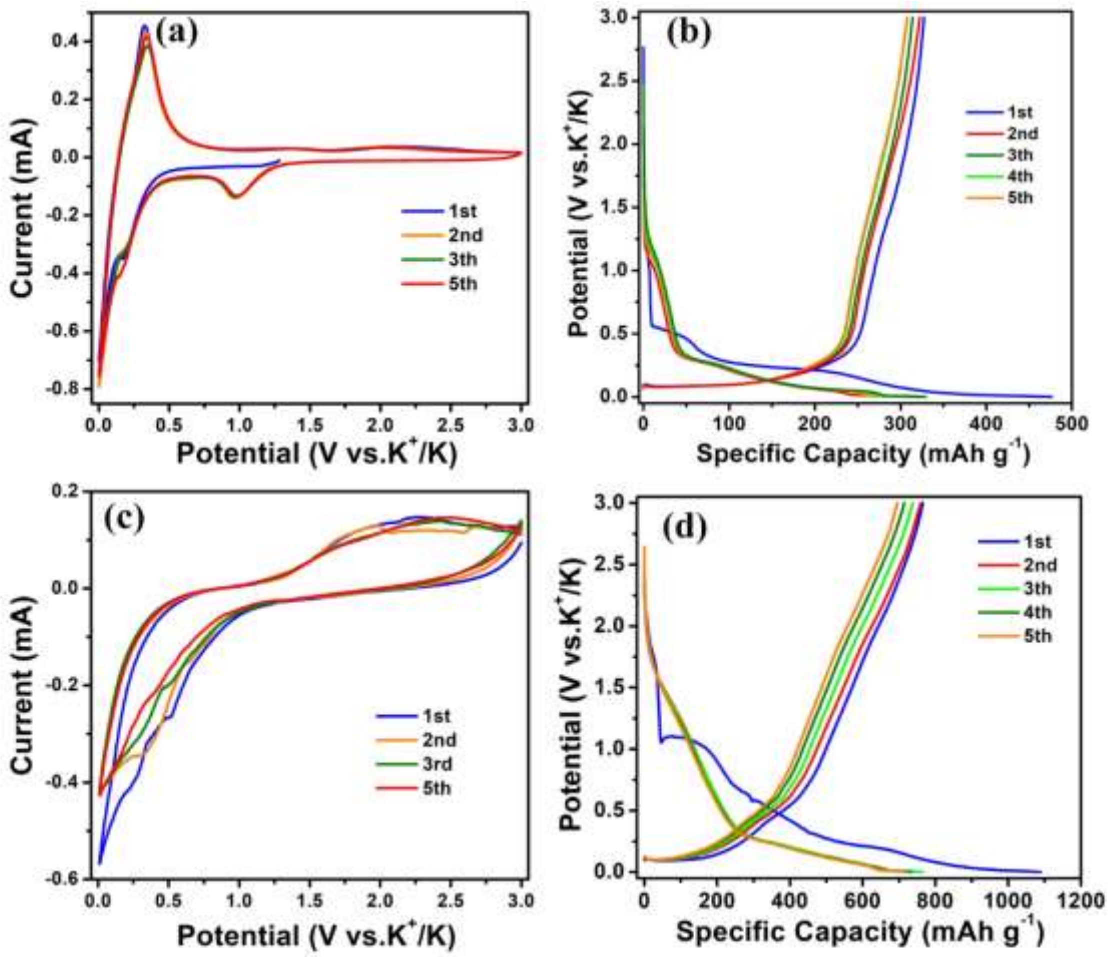

Figure S6. The CV and GCD curves of the NCO (a, b) and NCP (c, d) electrode, respectively.

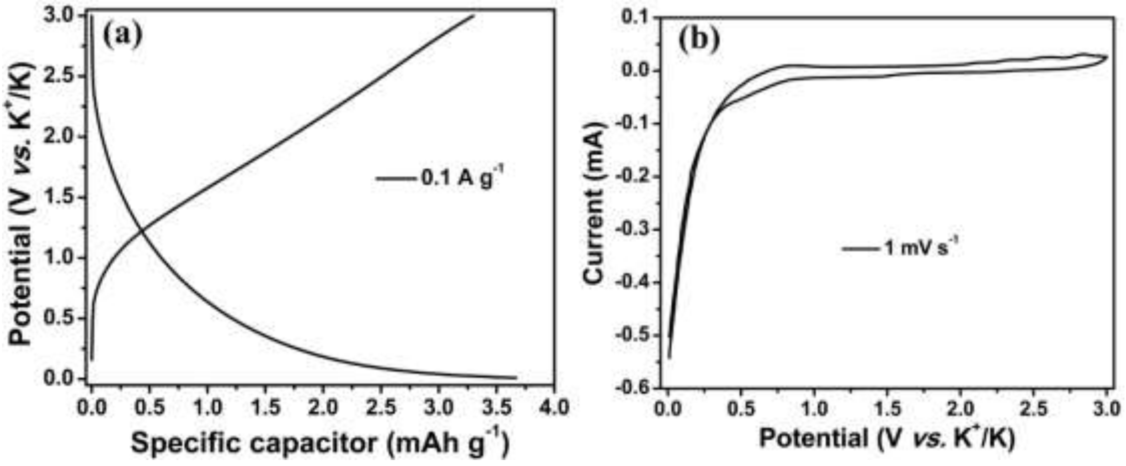

Figure S7. The electrochemical performance of the bare carbon cloth substrate in K ion half cells, the GCD curves at 0.1 A g^{-1}(a) and CV curves (b) at 1 mV s^{-1}.

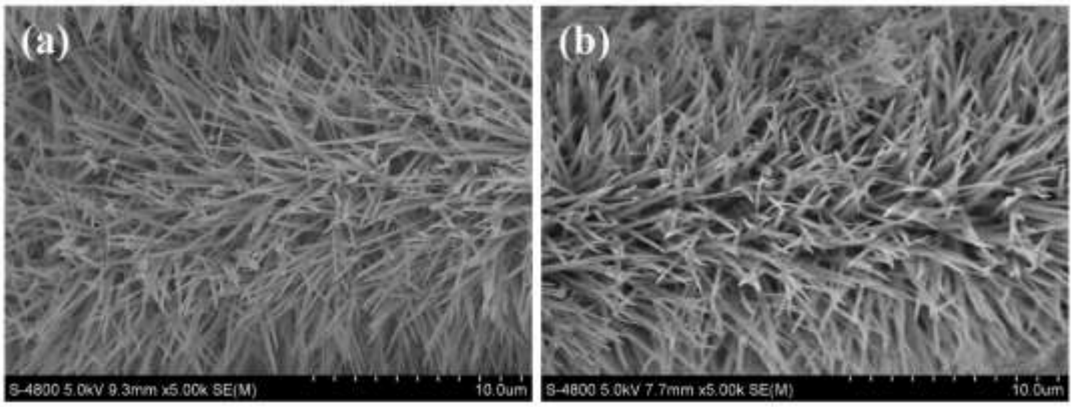

Figure S8. SEM images of the NCOP before (a) and after (b) 1,000 cycles at 1 A g^{-1}.

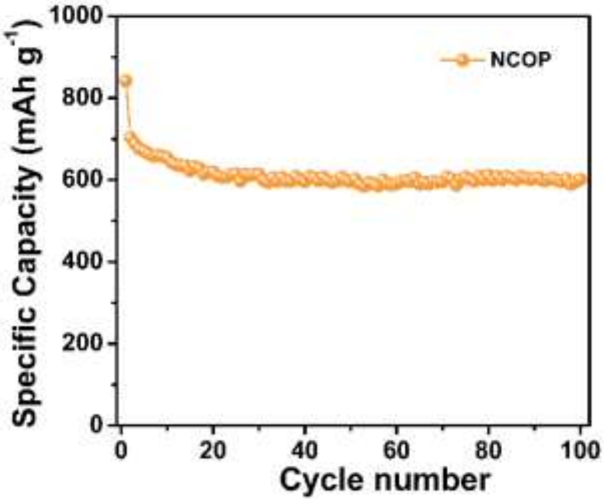

Figure S9. The cycling performance of the NCOP at 0.1 A g^{-1}

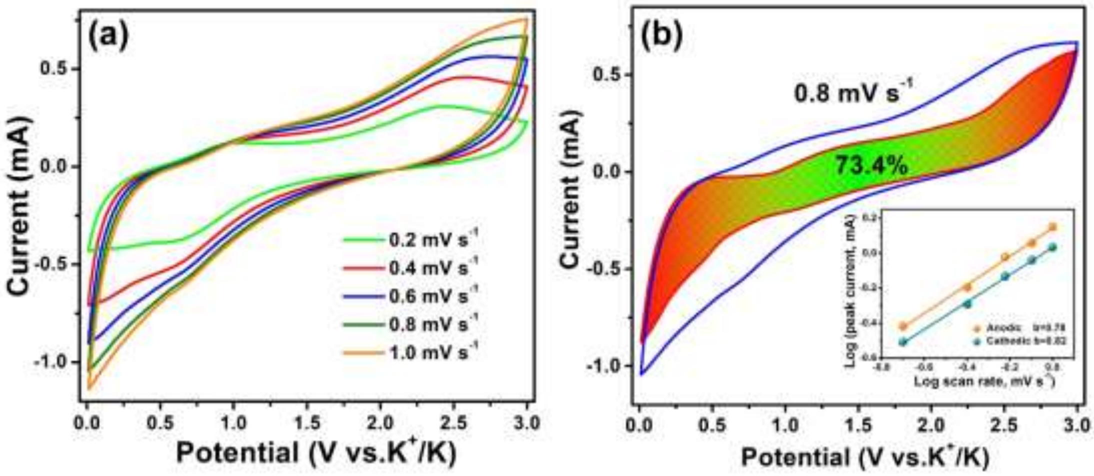

Figure S10. (a) CV curves of NCOP at scan rates range from 0.1 to 1.0 mV s^{-1}. (b) the contribution ratio of the capacitive capacities and diffusion-limited capacities at 1 mV

s^{-1}, insert show Linear relationship of log(i) vs. log(v).

As shown in the **Figure S10**, the b value of the andic and cathodic was calculated from the peak position at 0.58 V and 2.45 V, respectively.

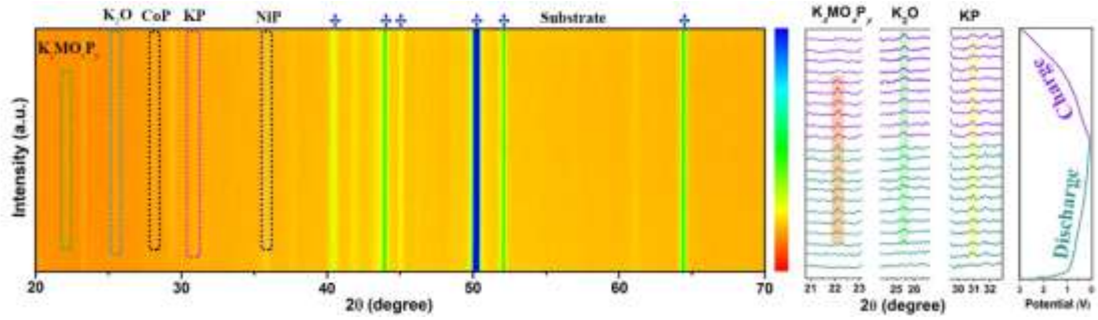

Figure S11. In-situ XRD patterns of NCOP electrode during discharge/charge process.

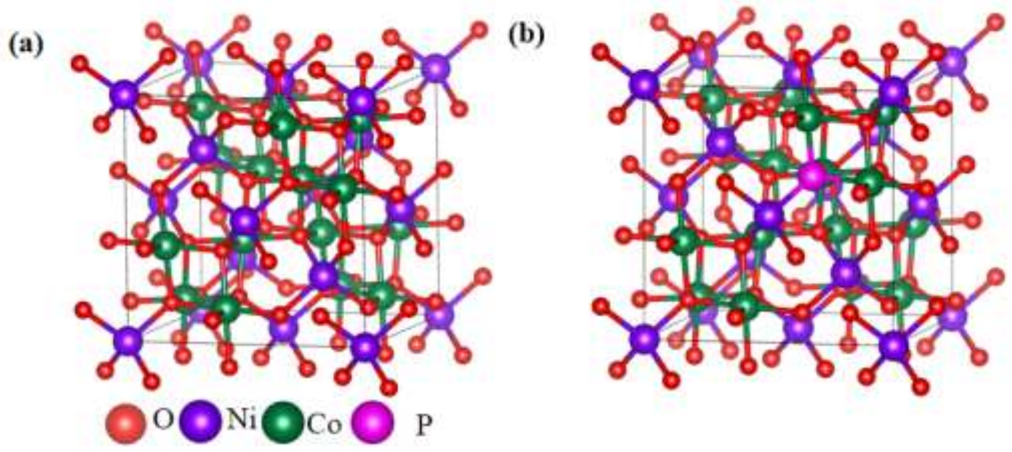

Figure S12. The calculation structure of the NCO and NCOP.

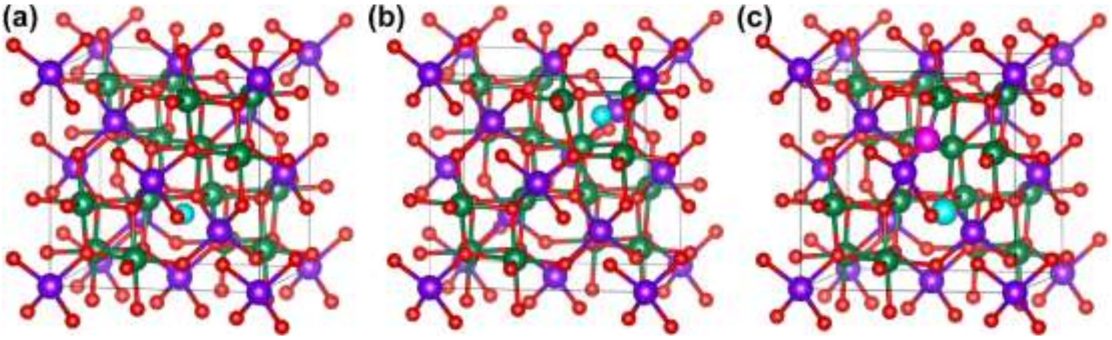

Figure S13. Structure diagram of K^+ embeddings in Ni_2CoO_4 at S1 (a) and S2 (b) sites,

the structure diagram of the K^+ embeddings in P-Ni_2CoO4 at S1 sites (c).

The structure diagram of K^+ embedded at different sites in Ni2CoO4 is selected. Figure S11a is site S1, Figure S11b is site S2, and the corresponding adsorption energy is -0.462 eV and -0.103 eV respectively. It can be seen from this that K+ is preferentially embedded at site S1, which is a gap site composed of Ni-Co-O. Figure S11c shows the structure of S1 site corresponding to potassium ion embedding in P-Ni_2CoO_4.

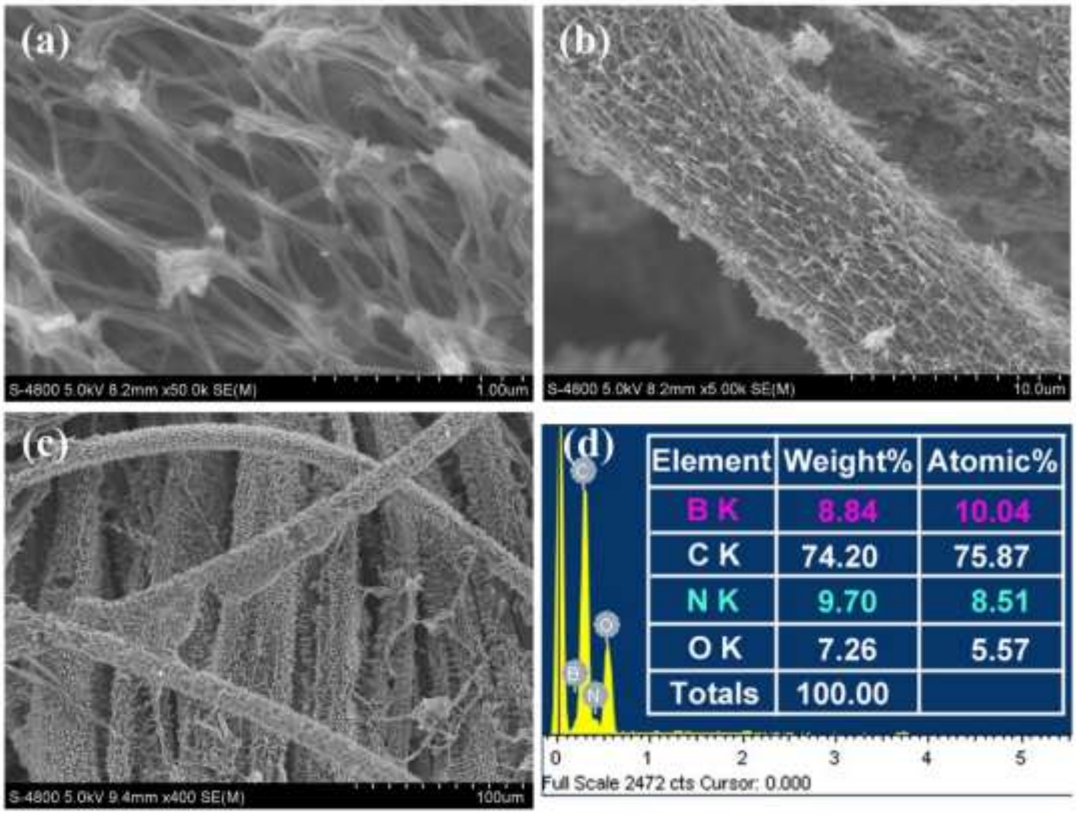

Figure S14. The morphology characterization of the BCN: the SEM image at high magnification (a) and low magnification (b, c); and the corresponding EDS result (d).

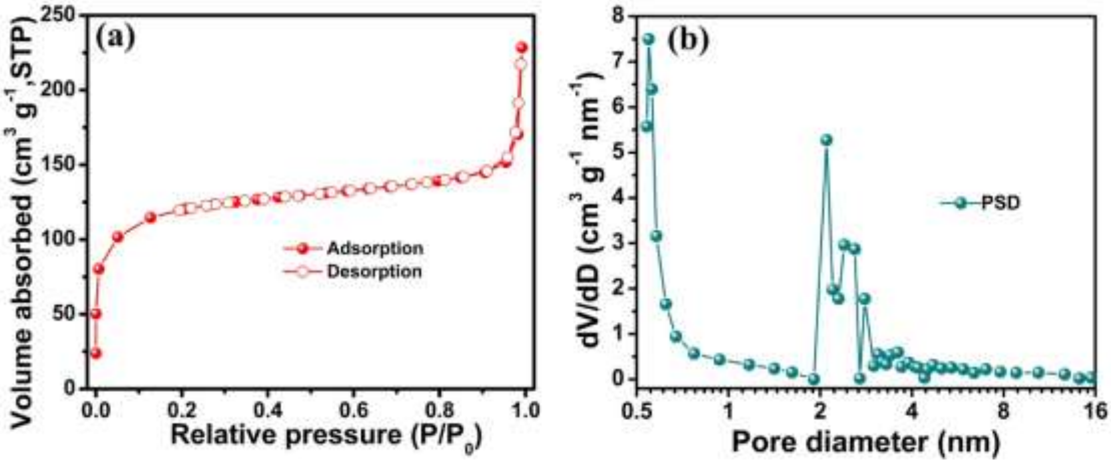

Figure S15. N_2 adsorption-desorption isotherm of the BCN, and the corresponding pore size distribution (b).

The pore size and the specific surface area of the BCN have been characterized used the N_2 adsorption–desorption isotherms (**Figure S15**). The Brunauer–Emmett–Teller (BET) specific surface area of 196.6 m^2 g^{-1}, and the pore size distribution revealing the pores size mainly concentrated in the micropore range (**Figure S15b**). These excellent properties give the nanocomposite better contact with the electrolyte/electrode and react thoroughly, thereby obtaining advanced energy storage performance.

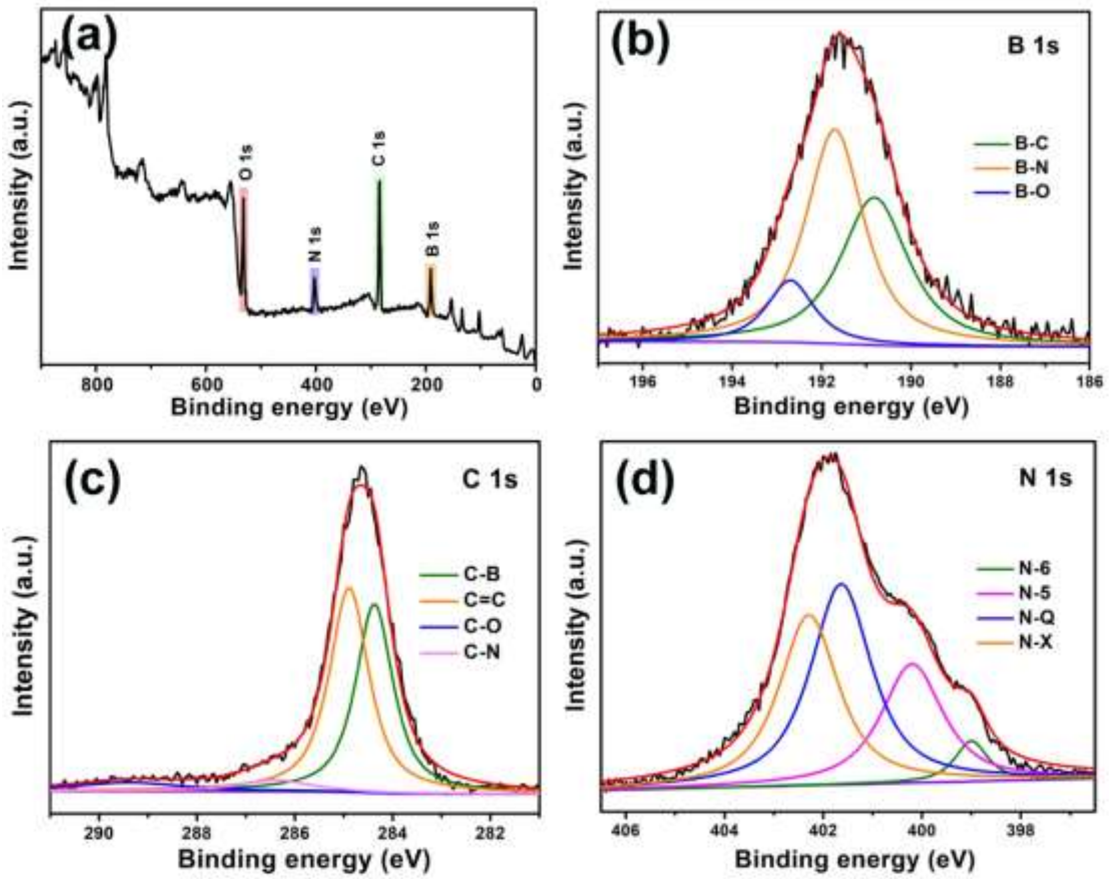

Figure S16. The XPS spectra of survey spectra of BCN (a), corresponding high-resolution B1s (b), C 1s (c), and N 1s (d) spectra, respectively.

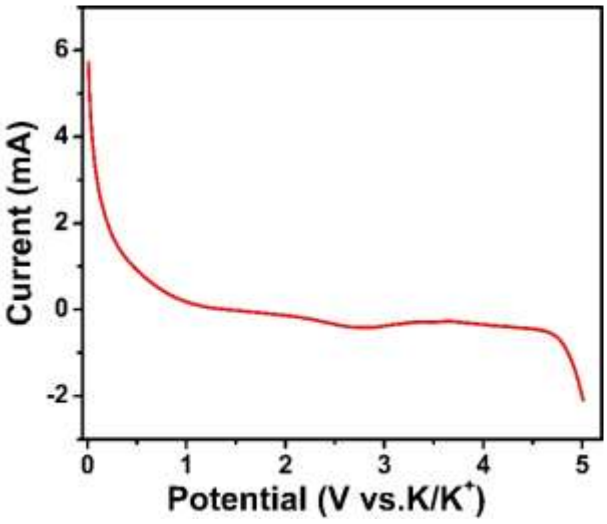

Figure S17. The LSV curves of the BCN materials in potassium-ion half-cell.

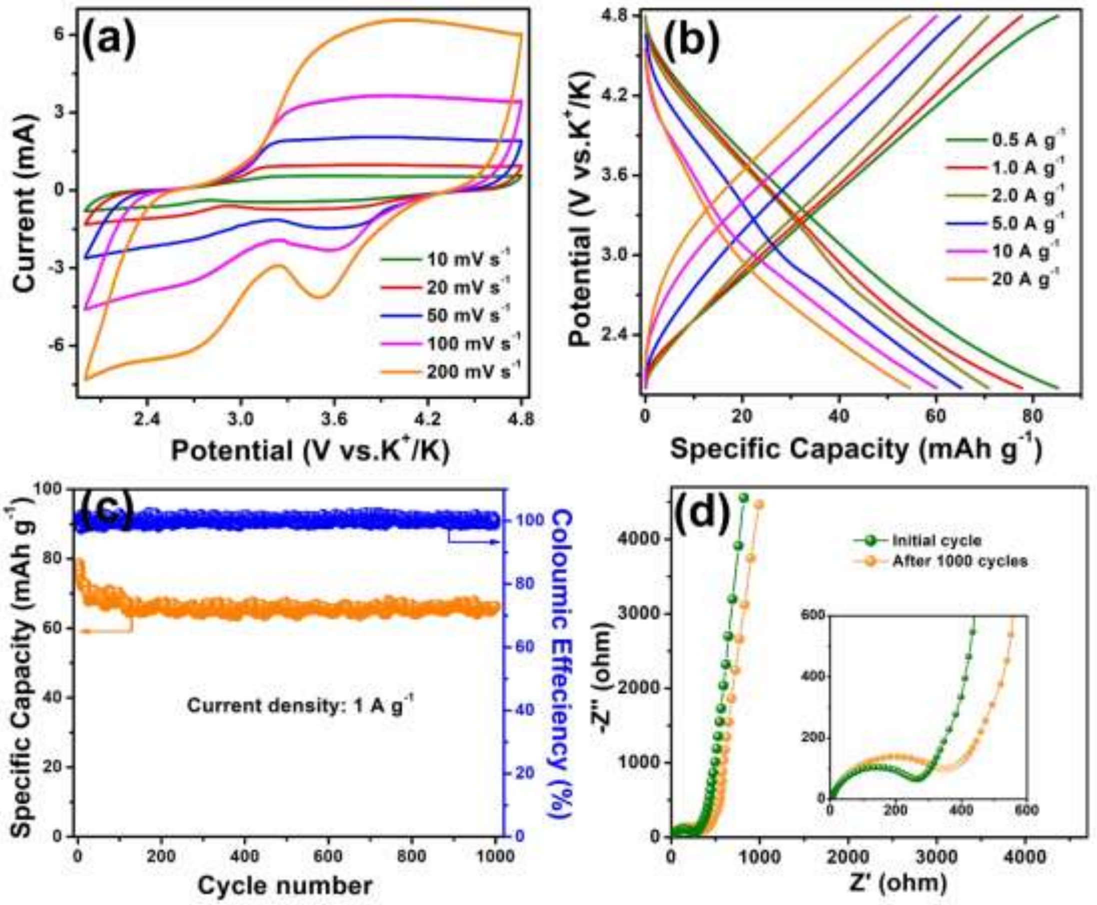

Figure S18. Electrochemical performance of BCN electrode: CV curves at increasing scan rate (a); GCD curves at an increasing current density (b); the cycling performance at 1 A g^{-1} (c), and EIS plots before and after 1000 cycles (d).

The CV curves (**Figure S18a**) of the BCN with some obvious redox peaks appears enhanced capacitance performance, which means the B/N co-doping strategy makes the carbon material not only with double layer contribution but also with fast surface pseudocapacitance contribution. The GCD curves of BCN electrode in **Figure S18b** reveal a linear shape, indicating that the electrode operates at an electrochemical double-layer capacitance. The BCN cathode delivers a capacity of 86 mAh g^{-1} at 0.5 A g^{-1}, even at 20 A g^{-1}, it also exhibits a capacity of 56 mAh g^{-1}. Furthermore, the electrode delivers outstanding cycling performance with 92.4% retention after 1000

cycles at 1.0 A g^{-1} (**Figure S18c**).

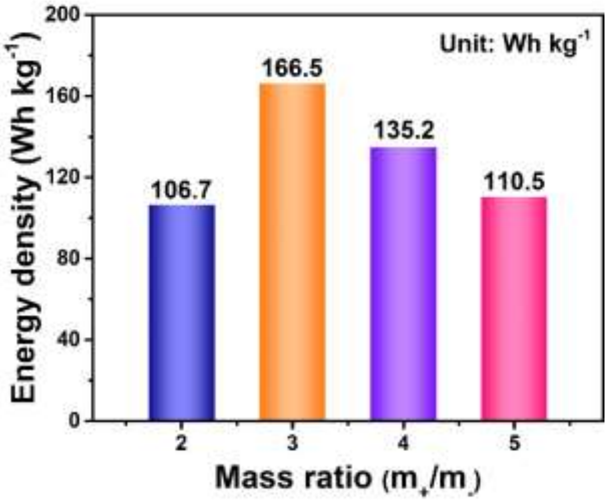

Figure S19. The specific capacity of NCOP//BCN hybrid devices with different mass ratios of positive vs. negative materials.

Table S1. The ICP-OES result of the NCOP powder material

Abbreviated chemical formula	Measured atomic ratio		
$NiCo_2O_3P$	Ni	Co	P
	1.0	2.23	0.78

Table S2. Comparison of KIB performance of NiCoOP composite with transition metal oxides-based electrode reported in the literatures.

Materials	Capacitance mAh g-1	Cycle performance	Reference
**NCOP**	0.1 A g^{-1} 721.5 mAh g^{-1}	1 A g^{-1}-332.5 mAh g^{-1}-500-62%	This work
**Fe-Ni-P**	50 mA g^{-1} 168 mAh g^{-1}	0.2 A g^{-1}-33 mAh g^{-1}-1000-70%	*Chem. Enginee. J* **2020,** *390,* 124515.
**C-WS_2@CNFs**	50 mA g^{-1} 319 mAh g^{-1}	2 A g^{-1}-158mAh g^{-1} -300-91%	*Energy Environ. Sci.* **2021,** *14,* 3184
**CoV_2O_6@GO**	100 mA g^{-1} 557 mAh g^{-1}	1 A g^{-1}-158.4 mAh g^{-1}-2000-60%	*Energy Storage Mater.* **2021,** *40,* 250.
**MoP@NC**	100 mA g^{-1} 256.1 mAh g^{-1}	4 A g^{-1}-255.2 mAh g^{-1}-800-89.9%	*Adv. Sci.* **2021,** *8,* 2004142
**MoP@CF**	100 mA g^{-1} 320 mAh g^{-1}	2 A g^{-1}-220 mAh g^{-1}-200-90%	*Small* **2019,** *16,* 1905301.
**Se_3P_4@C**	50 mA g^{-1} 1036.8mAh g^{-1}	1 A g^{-1}-220 mAh g^{-1}-300-79%	*Small* **2020,** *16,* 1906595.
**CoP@C**	50 mA g^{-1} 307 mAh g^{-1}	1 A g^{-1}-40 mAh g^{-1}-1000-99%	*Angew. Chem. Int. Ed.* **2020,** *59,* 5159.
**$NiCo_{2.5}S_4$@RGO**	50 mA g^{-1} 602 mAh g^{-1}	0.2 A g^{-1}-495 mAh g^{-1}-1900-82%	*Angew. Chem. Int. Ed.* **2019,** *58,* 14740.
**FeP/C**	50mA g^{-1} 289 mAh g^{-1}	0.05 A g^{-1}-289mAh g^{-1}-50-63.2%	ACS Appl. Mater. Interfaces, **2019,** *11,* 22364.
**SnO_2@CF**	500mA g^{-1} 371 mAh g^{-1}	1 A g^{-1}-231.7 mAh g^{-1}-4000-93.7%	*Energy Environ. Sci.* **2020,** *13,* 571.
**NiS_x@C**	100mA g^{-1} 415 mAh g^{-1}	2 A g^{-1}-116 mAh g^{-1}-8000-93.8%	*J. Mater. Chem. A* **2019,** *7,* 18932.
**Fe_3O_4/NPGF**	200mA g^{-1} 200 mAh g^{-1}	1 A g^{-1}-154.6 mAh g^{-1}-500-76.4%	*J. Mater. Chem. A* **2019,** *7,* 19430.
**FeP/GN**	100 mA g^{-1} 323 mAh g^{-1}	2 A g^{-1}-127mAh g^{-1}-2000-97.6%	*J. Mater. Chem. A* **2020,** *8,* 3369.
**Co_2P nanorods**	20 mA g^{-1} 374 mAh g^{-1}	0.2A g^{-1}-350mAh g^{-1}-5000-54%	*Nanoscale Horiz.* **2019,** *4,* 1394.
**SnS_2@rGO**	50 mA g^{-1} 448 mAh g^{-1}	0.2 A g^{-1}-205 mAh g^{-1}-300-73%	*Small* **2019,** *15,* 1804806.
**WS_2-NCN**	50 mA g^{-1} 469 mAh g^{-1}	0.5A g^{-1}-315 mAh g^{-1}-500-96.8%	*Matter* **2019,** *1,* 893.
**$MoSe_2$/N-C**	100 mA g^{-1} 300 mAh g^{-1}	0.1A g^{-1}-258.2mAh g^{-1}-300-100%	*Adv. Energy Mater.* **2018,** 1801477

Table S3. The K ion is embedded in Ni_2CoO_4 and P-Ni_2CoO_4, corresponding the lattice parameters, volume change rate and binding energy.

Structure	Lattice parameters	Volume(Å^3)	ΔV (Å^3)	Eb (eV)
**Ni_2CoO_4**	a=8.1513, b=8.1513, c=8.1513	541.363	--	--
**K-Ni_2CoO_4**	a=8.5788, b=8.5535, c=8.4336	626.185	13.5%	-0.462
**P-Ni_2CoO_4**	a=8.4911, b=8.4911, c=8.4817	611.520	--	--
**K-P-Ni_2CoO_4**	a=8.6613, b=8.5613, c=8.5716	635.610	3.7%	-0.861

Table S4. Comparison of KIHCs performance of NiCoOP with currently reported in the literatures.

Materials	voltage window (V)	Capacitance F g^{-1}	Cycle performance	Power density kW kg^{-1}	Energy density Wh kg^{-1}	Reference
NCOP//BCN	0.01-4.5	0.1 A g^{-1}- 59.3 F g^{-1}	5.0 A g^{-1}- 10000- 86.5%	22.5	166.5	This work
Co_2P@rGO //AC	1.0-4.0	0.02 A g^{-1}- 11.75 F g^{-1}	1 A g^{-1}- 1000-68%	4.265	87	Nanoscale Horiz. **2019,** *4*, 1394.
NCHS//WS_2@C	0.5-4.2	0.05 A g^{-1}- 57.8mAhg^{-1}	0.2 A g^{-1}- 2000-	0.235	103.4	*Matter* **2019,** *1*, 893.
CoP@NPC//MBC	0.01-4.0	0.5 A g^{-1}- 112.5 F g^{-1}	1 A g^{-1}- 83%-3000	11	180.3	*J. Mater. Chem. A* **2021,** *9*, 16028
CoV_2O_6@GO//AC	0.5-4.0	0.05 A g^{-1}- 75 F g^{-1}	1 A g^{-1}- 96.8%- 3000	22.5	78.2	*Energy Storage Mater.* **2021,** *40*, 250.
MoP@NC//AC	0.01-4.0	0.05A g^{-1}- 32 F g^{-1}	-	20.42	69.7	*Adv. Sci.* **2021,** *8*, 2004142.
TiO_2/NC//AC	0.01-3.8	0.05 A g^{-1}- 60 mAh g^{-1}	2.5 A g^{-1}- 30000- 100%	5.500	108.6	*Chem. Engine. J.* **2021,** *417*, 127977.
C-WS_2//ACNF	0.01-4	0.2 A g^{-1}- 65 mAh g^{-1}	4 A g^{-1}- 5500- 81.4%	12.6	180.4	*Energy Environ. Sci.* **2021,** *14*, 3184.
CNS//FCDAC	0.01-4.2	0.5 A g^{-1}- 144mAh g^{-1}	2 A g^{-1}- 5000-80%	21	149	*Adv. Energy Mater.* **2019,** *9*, 1803894.
MoSSe//AC	0.01-3.5	0.2A g^{-1}- 32 F g^{-1}	2 A g^{-1}- 6000- 91.3%	15.4	77.4	*J. Mater. Chem. A* **2020,** *8*, 13946.
CoP@rGO//AC	1.0-4.0	0.02 A g^{-1}- 153 F g^{-1}	2 A g^{-1}- 1000-68%	4.26	87	*Nanoscale Horiz.* **2019,** *4*, 1394.
MoP@NC-1//AC	0.01-4.0	0.05A g^{-1}- 32.5 F g^{-1}	1 A g^{-1} - 2000- 61.2%	2.04	69.7	*Adv. Sci.***2021,** *8*, 202004142
CBC@G//ACBC	0.01-4.0	0.05A g^{-1}- 91 mAh g^{-1}	5A g^{-1}- 5000- 81.5%	22	172	*Adv. Sci. 2020, 7, 2001681*
AC//S-N-PCNs	0.01-4.0	0.05 A g^{-1}- 95 F g^{-1}	1 A g^{-1}- 3000- 86.4%	2.136	197	*Adv Energy Mater* **2019,** *9, 1901533.*
P/O-PCS//AC	0.01-4.0	0.05 A g^{-1}- 30 F g^{-1}	5 A g^{-1}- 30000- 94.5%	1.38	158	*Adv. Funct. Mater.* **2021**, *31*, 2102060
SGN-900 //NGN	0-4.2	1 A g^{-1}-100 F g^{-1}	2 A g^{-1}- 6000-92%	14.6	112	*ACS Nano* **2021,** *15*, 1652
WS2@CNFs//ACNFs	0-4.0	0.2A g^{-1}- 77.2 F g^{-1}	4 A g^{-1}- 5500- 81.4%	12.6	180.4	*Energy Environ. Sci.*, **2021,***14*, 3184.

BPCS//NPC	0.01-4.5	0.5A g^{-1}-86.1 F g^{-1}	2 A g^{-1} -10000-80.8%	30	167	*ACS Nano* **2020,** *14,* 4938.
Ti_3C_2//HPAC	1.0-4.0	0.05 A g^{-1}-45 F g^{-1}	1 A g^{-1}-10000-100%	7.02	98.4	*Adv. Funct. Mater.* **2020,** *30,* 2005663.
Ti3C2Tx//AC	1.0-4.0	0.05 A g^{-1}-32 F g^{-1}	1 A g^{-1}-10000-101%	8.7	163	*Angew.Chem. Int.Ed.* **2021,** *60,*26246.
APN-HPCNF//PN-HPCNF	0.01-4.0	0.05 A g^{-1}-93.5 F g^{-1}	1 A g^{-1}-8000-82.3%	7.56	191	*Energy Environ. Sci., 2020, 13, 2431-2440*
HCMB//AC	2.5-4.5	0.1 A g^{-1}-78 F g^{-1}	20 mV s-1-1000-90%	17.5	152	*Energy Environ. Sci., 2021, DOI: 10.1039/D1EE03214C*
BSH//AC	0.01-4.0	0.05 A g^{-1}-65.5 F g^{-1}	0.35 A g^{-1}-1000-71.4%	0.6	120	*Adv. Mater.* **2018,** *30,* 1800804.
$NbSe_2$/NSeCNFs//AC	0.01–3.8	0.05 A g^{-1}-88 F g^{-1}	2 A g^{-1}-10000-83%	4.0	145	*Adv. Funct. Mater.* **2020,** *30,* 2004247.
